# *Lactobacillus paracasei*-derived extracellular vesicles attenuate the intestinal inflammatory response by augmenting the endoplasmic reticulum stress pathway

**DOI:** 10.1038/s12276-019-0359-3

**Published:** 2020-03-02

**Authors:** Ji Hyun Choi, Chang Mo Moon, Tae-Seop Shin, Eun Kyoung Kim, Andrea McDowell, Min-Kyung Jo, Yang Hee Joo, Seong-Eun Kim, Hye-Kyung Jung, Ki-Nam Shim, Sung-Ae Jung, Yoon-Keun Kim

**Affiliations:** 1 Department of Internal Medicine, College of Medicine, Ewha Womans University, Seoul, Republic of Korea; 2 Tissue Injury Defense Research Center, Ewha Womans University, Seoul, Republic of Korea; 3 MD Healthcare Inc., Seoul, Republic of Korea

**Keywords:** Acute inflammation, Chronic inflammation

## Abstract

*Lactobacillus paracasei* is a major probiotic and is well known for its anti-inflammatory properties. Thus, we investigated the effects of *L. paracasei*-derived extracellular vesicles (LpEVs) on LPS-induced inflammation in HT29 human colorectal cancer cells and dextran sulfate sodium (DSS)-induced colitis in C57BL/6 mice. ER stress inhibitors (salubrinal or 4-PBA) or CHOP siRNA were utilized to investigate the relationship between LpEV-induced endoplasmic reticulum (ER) stress and the inhibitory effect of LpEVs against LPS-induced inflammation. DSS (2%) was administered to male C57BL/6 mice to induce inflammatory bowel disease, and disease activity was measured by determining colon length, disease activity index, and survival ratio. In in vitro experiments, LpEVs reduced the expression of the LPS-induced pro-inflammatory cytokines IL-1α, IL-1β, IL-2, and TNFα and increased the expression of the anti-inflammatory cytokines IL-10 and TGFβ. LpEVs reduced LPS-induced inflammation in HT29 cells and decreased the activation of inflammation-associated proteins, such as COX-2, iNOS and NFκB, as well as nitric oxide. In in vivo mouse experiments, the oral administration of LpEVs also protected against DSS-induced colitis by reducing weight loss, maintaining colon length, and decreasing the disease activity index (DAI). In addition, LpEVs induced the expression of endoplasmic reticulum (ER) stress-associated proteins, while the inhibition of these proteins blocked the anti-inflammatory effects of LpEVs in LPS-treated HT29 cells, restoring the pro-inflammatory effects of LPS. This study found that LpEVs attenuate LPS-induced inflammation in the intestine through ER stress activation. Our results suggest that LpEVs have a significant effect in maintaining colorectal homeostasis in inflammation-mediated pathogenesis.

## Inflammatory bowel disease: Vesicles from gut bacteria quell inflammation

Tiny vesicles released by a bacterial species found in the human gut can reduce symptoms of inflammatory bowel disease (IBD) and prevent disease progression. People with IBD have a decreased abundance of *Lactobacilli* bacteria in their gut, creating an imbalance that perpetuates the disease. Replenishment of this bacteria may become a valuable therapy. Chang Mo Moon at Ewha Womans University, Yoon-Keun Kim at MD Healthcare, both in Seoul, South Korea, and co-workers demonstrated how extracellular vesicles (EVs) released by *Lactobacilli paracasei* can actively prevent bowel inflammation. These EVs contain a mixture of proteins, nucleic acids and other biomolecules. The team administered EV to cultured human colorectal cancer cells and to mice with induced colitis. The EVs decreased pro-inflammatory protein activity and boosted levels of protective cellular membrane proteins via augmenting ER stress pathway.

## Introduction

The human microbiome has been extensively investigated for its role in the prevention and treatment of inflammatory bowel disease (IBD)^1,2^. Humans are populated with trillions of bacteria that are located primarily in the gastrointestinal tract and have vast, complex immunomodulatory effects on their host. In comparison to healthy subjects, patients suffering from IBD are generally known to have decreased abundance of Lactobacilli populating their GI tract, suggesting that replenishment of Lactobacilli in the gut may be an appropriate therapeutic target for IBD patients^3^. Healthy balance of the gut microbiota is essential for the normal development and function of the immune system, beginning with initial bacterial colonization initiated at birth through the vaginal canal. Interestingly, studies have shown that offspring delivered by cesarean section rather than vaginal delivery have disrupted bacterial colonization associated with decreased *Lactobacillus* spp. and increased rates of IBD^4,5^. Therefore, a clear link has been shown to exist between IBD and the vaginal flora that contribute to the early development of the intestinal microbiota, which is dominated by *Lactobacillus* spp. One such species, *Lactobacillus paracasei*, has shown particular promise as a probiotic candidate through its ability to decrease the secretion of pro-inflammatory cytokines, increase the production of anti-inflammatory cytokines^6^, increase immunomodulatory control^7,8^, and decrease IBD symptom severity^9,10^.

While links between microbiota composition and IBD have been established, the specific underlying molecular mechanisms of action remain unclear. An often overlooked bacterial function is the release of extracellular vesicles (EVs), nanometer-sized vesicles composed of lipid bilayer membranes designed to transport diverse biomolecules. Due to their ability to transverse epithelial cells, bacterial EVs can be found in both the extracellular space and various biological fluids and have a crucial role in the transport of microRNAs (miRNAs), messenger RNAs (mRNAs), and proteins from parental cells to recipient cells^11,12^. EVs play an important role in cell-to-cell communication and, consequently, the regulation of cellular physiology and pathophysiology. Indeed, EVs in biological fluids have been found to play a role in the body’s immunological mechanisms^13–16^. These findings imply that circulating microbial EVs may directly influence the health-promoting functions of probiotics and represent a primary molecular mechanism of the immunomodulatory effects of probiotics. This is supported by previous reports that *Lactobacillus*-derived EVs alleviate inflammatory responses by regulating cytokine production^17,18^.

The endoplasmic reticulum (ER) is an organelle that is responsible for protein modification and the maintenance of intracellular calcium homeostasis^19^. The accumulation of unfolded proteins and imbalanced calcium homeostasis can disturb ER functions, causing ER stress. ER stress activates three ER membrane proteins known to induce adaptive immune responses: serine/threonine kinase PKR-like ER kinase (PERK), inositol-requiring enzyme 1 (IRE1), and activating transcription factor (ATF6)^20,21^. When ER disturbance is too severe for recovery, CHOP activation triggers ER stress-associated apoptosis to eliminate damaged cells^22,23^. ER stress has been implicated in many diseases, including obesity, diabetes, inflammatory diseases, and neurodegenerative disorders^24–27^. ER stress is also a mechanism of action for anti-inflammatory agents^28^.

In the present study, we aimed to investigate whether EVs from *L. paracasei*, a newly isolated lactic acid bacterium in the human body, mediates anti-inflammatory actions. Furthermore, we attempted to understand how ER stress impacts the anti-inflammatory effects of *L. paracasei*-derived EVs (LpEVs) in in vitro and in vivo experimental colitis models.

## Materials and methods

### Materials

Fetal bovine serum (FBS), Dulbecco’s modified Eagle medium (DMEM), and other cell culture products were purchased from Life Technologies (Grand Island, NY, USA). Lipopolysaccharide (LPS), 4-phenylbutyric acid (4-PBA), salubrinal, and 2,3-diaminonaphthalene (2,3-DAN) were obtained from Sigma-Aldrich (St. Louis, MO, USA). TRIzol reagent and lipofectamine siRNA transfection reagent were purchased from Invitrogen (Carlsbad, CA, USA). Small interfering RNAs (siRNAs) against scrambled control and CHOP were obtained from M Biotech (Seoul, Korea). Antibodies against COX-2, CHOP, and ATF6α (p90) were purchased from Santa Cruz Biotechnology (Santa Cruz, CA, USA). Antibodies against phospho-PERK (p-PERK), nuclear factor κB (NFκB), and iNOS were purchased from BioLegend (San Diego, CA, USA). Antibodies against phosphor-IRE1α were purchased from Abcam (Cambridge, MA, USA). Antibodies against phospho-IκB (p-IκB) and IκB were purchased from Cell Signaling Technology (Beverly, MA, USA). An enhanced chemiluminescence (ECL) system was obtained from GE Healthcare Life Sciences (Chicago, Illinois, USA).

### Ethics statement

This study was conducted strictly according to the recommendations in the Guide for the Care and Use of Laboratory Animals of the National Institute of Health. The experimental protocols were approved by the Institutional Animal Care and Use Committee at Ewha Womans University, Seoul, Republic of Korea. All animal experiments were designed to minimize mouse sacrifice.

### A mouse model of dextran sulfate sodium-induced colitis

Seven-week-old male C57BL/6 mice (Orient Bio Inc., Seongnam, Korea) were maintained on a 12-h day/night cycle under specific pathogen-free conditions. The mice were fed a standard diet and water until the age of 8 weeks and divided into three groups (six mice in each group). For the acute colitis mouse model, mice were provided dextran sulfate sodium (DSS) (36–50 kDa; MP Biomedicals, LLC, Illkirch, France) in drinking water at a concentration of 2% (weight/volume) for 5 days. The healthy control mice were provided drinking water without DSS. A total of 5 mg of *L. paracasei* EVs suspended in phosphate-buffered saline (PBS) were administered to the LpEVs+DSS group from day 0 to day 12 by oral gavage. The mice were sacrificed on day 13 to investigate clinical pathology and parameters. qRT-PCR was conducted to analyze the mRNA expression levels of cytokines. Mouse primers for each gene are shown in Supplementary Table 1.

### In vivo fluorescence imaging

LpEVs (µg) were incubated with 5 μM Cy7 mono NHS ester (GE Healthcare, Little Chalfont, UK) for 1 h at 37 °C. Cy7 mono NHS ester-labeled LpEVs were isolated using ultracentrifugation. Then, Cy7-labeled LpEVs (10 μg of total protein) were administered by gavage to the mice, which had been fasted overnight. At the indicated time point, whole-body images were obtained at a wavelength of 780–800 nm using a Davinch-Invivo system (Davinch-Invivo Fluoro Chemi, Korea). After whole-body imaging, the mice were sacrificed, and Cy7 fluorescence in the dissected organs was quantified.

### Measurement of disease activity index and colon length

To evaluate the disease activity index (DAI), body weight, stool consistency, and stool blood were monitored and recorded daily. DAI was determined by calculations established previously^29^. Mice from the DSS group that had died received a DAI of 12 points. After mouse sacrifice, the colons were extracted, and the colon length between the ileocecal junction and the rectum was measured. To extract protein, the colon was stored at −80 °C. For qPCR, the colon was subjected to RNAlater Stabilization Solution (20 mM EDTA, 25 mM sodium citrate tribasic dihydrate, and 70% ammonium sulfate) at 4 °C overnight and used for total RNA isolation.

### Preparation of *L. paracasei-*derived EVs

Isolation and purification of EVs was performed as previously described^30^. *L. paracasei* was isolated from the vaginal discharge of a woman from a previous study at Chung-Ang University (IRB No. 10-089-12-24). *L*. *paracasei* was cultured in MRS broth (MB cell, CA, USA) for 18 h at 37 °C with gentle shaking (150 r.p.m.). When the optical density of the culture at 600 nm reached 1.0, the bacteria were pelleted at 10,000 × *g* for 20 min, and the resulting supernatant was passed through a 0.22-μm bottle-top filter (Corning, NY, USA) to remove any remaining cells. The filtrate was concentrated with a MasterFlex pump system (Cole-Parmer, IL, USA) using a 100-kDa Pellicon 2 Cassette filter membrane (Merck Millipore, MA, USA) and subsequently passed through a 0.22-μm bottle-top filter. EVs were obtained from the resulting filtrate by ultracentrifugation at 150,000 × *g* for 3 h at 4 °C. The protein concentration was measured by the BCA assay (Thermo Fisher Scientific, MA, USA), and the collected fractions of EVs were stored at −80 °C until use.

### Heat inactivation of *L. paracasei*

*L. paracasei* was cultured and heat inactivated by placement in a 70 °C water bath for 1 h. After heat inactivation, the bacteria were pelleted at 10,000 × *g* for 20 min, and the supernatant was discarded. The inactivated bacterial pellet was resuspended in PBS. The protein concentration was measured by the BCA assay (Thermo Fisher Scientific, MA, USA).

### Genome sequencing and de novo assembly and annotation

*L. paracasei* cells cultivated in MRS broth (Difco) were harvested in the middle phase of logarithmic growth. PacBio SMRT whole-genome sequencing was conducted utilizing a PacBio RSII sequencer, producing 151,050 adapter-trimmed reads (subreads) with an average read length of approximately 7040 bp. De novo assembly was performed with the RS HGAP Assembly v3.0 system utilizing the SMRT Portal 2.3 software, and the genome was annotated using Prokka Pipeline (Prokka v1.12b).

### Phylogenetic study

Reorganization of evolutionary affiliations was conducted at the National Center for Biotechnology Information (NCBI)-BLAST. 16S ribosomal RNA (rRNA) sequence data were acquired from GenBank (*Lactobacillus casei* NCDO161, *Pediococcus claussenii* ATCC BAA-344, *Oenococcus oeni* 59b, *Lactobacillus. perolens* L532, *Lactobacillus coryniformis* subsp*. Torquens* 30, *Lactobacillus rhamnosus* JCM 1136, *L. rhamnosus* NBRC 3425, *L. paracasei* ATCC 25302, *L. paracasei* NBRC 15889, *L. paracasei* R094, *L paracasei* subsp*. Tolerans* NBRC 15906, *L. brantae* SL1108) to construct a phylogenetic tree among *Lactobacillus* strains.

### Transmission electron microscopy image analysis

Purified EVs were diluted to a concentration of 50 µg/mL in PBS, and 10 µL of the diluent was placed on a 300-mesh copper grid (EMS, Hatfield, PA, USA) and stained with 2% uranyl acetate for 5 min. The samples were visualized with an H-7650 TEM (Hitachi Ltd., Berkshire, UK).

### Dynamic light scattering

Purified EVs were diluted to 1 µg/mL with PBS, and the size distribution of EVs was measured using a Zetasizer Nano ZS instrument (Malvern Instruments, Worcestershire, UK) and Dynamic V6 Software 32.

### Cell culture

RAW 264.7 murine macrophages and HT29 human colorectal cancer cells were purchased from Korean Cell Line Bank (Seoul, Korea). HT29 cells were cultured in DMEM supplemented with 10% FBS, 100 U/mL penicillin, and 100 µg/mL streptomycin. Exponentially growing cultures were incubated in a humidified atmosphere of 5% CO_2_ at 37 °C. For treatment, the cells were serum-starved for 3 h and incubated with LpEVs, LPS, or other drugs for the indicated times.

### Analysis of cell viability

RAW 264.7 cells were treated with LpEVs (0.1, 1, 10 µg/mL) for 12 h. After 12 h, cell viability was determined by a cell viability assay kit (DoGen Bio, Seoul, Korea). The reagent was added to the cells according to the manufacturer’s protocol, and the cells were incubated at 37 °C for 3 h. Then, the optical density was read at 450 nm.

### Enzyme-linked immunosorbent assay

RAW 264.7 cells were pretreated with LpEVs (0.1, 1, 10, 50 µg/mL) or heat-inactivated bacterial pellets (0.1, 1, 10, 50 µg/mL) for 12 h and then stimulated by *Escherichia coli* EVs (1 µg/mL) for 12 h. The supernatant was centrifuged, collected, and analyzed by enzyme-linked immunosorbent assay (ELISA). ELISA was performed according to the manufacturer’s protocol (R&D Systems, Minneapolis, MN, USA). All reagents for ELISA were purchased from R&D Systems.

### RNA interference (siRNA)

siRNA transfection was performed using Lipofectamine siRNA Transfection Reagent. HT29 cells were plated overnight in six-well plates, and the medium was replaced with 1 mL of serum-free DMEM before transfection. The scrambled control or CHOP siRNA duplexes were incubated with 5 µL of siRNA transfection reagent for 5 min at room temperature; the mixture was then added to these cells. After 12 h, 1 mL of DMEM containing 10% FBS was added to each well. For experiments, cells were transfected with siRNA for 24 h and then treated with LPS with or without LpEVs for 24 h.

### Western blot analysis

Cells were pretreated with LpEVs for 12 h, treated with LPS treatment for the indicated times, and washed twice with ice-cold PBS. The cells were lysed in RIPA lysis buffer containing 50 mM Tris-HCl (pH 7.4), 150 mM NaCl, 1.0% NP-40, 0.5% sodium deoxycholate, 0.1% sodium dodecyl sulfate (SDS), 50 mM NaF, 0.1 mM phenylmethanesulfonyl fluoride (PMSF), and 0.5% protease inhibitor cocktail. The whole-cell lysates were centrifuged at 12,000 × *g* for 10 min at 4 °C to remove cellular debris and subjected to the BCA protein assay to determine the protein concentration. Cell lysates containing equal amounts of protein (40 µg) were resolved by 8–12% SDS-polyacrylamide gel electrophoresis (SDS-PAGE) and then analyzed by western blot analysis. Each blot was subjected to blocking with 5% skim milk in Tris-buffered saline with 0.05% Tween 20 (TBST) for 1 h at room temperature, treated with primary antibodies (1:2000) in TBST overnight at 4 °C, washed three times with TBST (10 min per wash), and incubated with HRP-conjugated secondary IgG (1:2000) in TBST for 2 h at room temperature. The protein expression was visualized with an ECL detection system according to the manufacturer’s protocols.

### Nuclear and cytoplasmic extraction

Cells were suspended in hypotonic buffer (10 mM 4-(2-hydroxyethyl)-1-piperazineethanesulfonic acid [HEPES] (pH 7.9), 1.5 mM MgCl_2_, 10 mM KCl, 0.2 mM PMSF, 0.5 mM dithiothreitol [DTT], and 10 μg/mL aprotinin) and 10% NP-40. After vortexing, the cell lysates were centrifuged at 4000 r.p.m. for 5 min to separate the supernatant (cytosolic fraction) and pellet (including the nucleus). The pellet was resuspended in high-salt buffer (20 mM HEPES (pH 7.9), 25% (w/v) glycerol, 0.4 M KCl, 1.5 mM MgCl_2_, 0.2 mM EDTA, 0.2 mM PMSF, and 0.5 mM DTT) and subjected to centrifugation at 14,000 r.p.m. for 5 min to collect the supernatant (nuclear fraction).

### Assessment of nitric oxide production

To measure the nitric oxide (NO) concentration, 2,3-diaminonaphthalene (DAN) was used according to previously described methods. Cells were treated with LPS alone or pretreated with LpEVs for the indicated times. The cells were washed twice with PBS, resuspended in 158 µM DAN/0.62 N HCl, and then incubated at 37 °C for 15 min. After 15 min, 2.8 N NaOH was added to stop the reaction between DAN and NO_2_. The fluorescence intensity was determined by flow cytometry using excitation and emission wavelengths of 365 and 450 nm, respectively. At least 10,000 events were analyzed per sample, and each sample was analyzed in duplicate.

### Total RNA extraction and qRT-PCR

To determine the effect of LpEVs on the expression of inflammatory cytokine genes, quantitative reverse transcriptase polymerase chain reaction (qRT-PCR) was performed. Total RNA was extracted from cultured cells (in vitro) and colorectal tissues (in vivo) using TRIzol reagent according to the manufacturer’s instructions. The quantity and purity of the total RNA samples were measured utilizing ultraviolet spectroscopy (NanoDrop 2000 Spectrophotometer; Thermo Fisher Scientific, Waltham, MA, USA). Complementary DNA (cDNA) was synthesized using M-MLV reverse transcriptase, and polymerase chain reaction (PCR) was performed. The PCR primers for each gene are shown in Supplementary Table 2.

To measure mRNA expression, real-time PCR analysis was performed with the SYBR Green qPCR Mastermix kit (Applied Biosystems) according to the manufacturer’s instructions. Samples were subjected to the QuantStudio 3 Real-time PCR system (Applied Biosystems). qPCR was performed under the following conditions: 30 s at 94 °C for denaturation, 5 s at 94 °C for annealing, and 30 s at 60 °C for extension. β-Actin was used as a loading control.

### Statistical analysis

All data are shown as the mean ± standard deviation of at least three independent experiments. Statistical comparisons were carried out by using one-way analysis of variance followed by Tukey’s post hoc test (GraphPad Prism version 5.0, GraphPad Software Inc. San Diego, CA, USA). *P* values < 0.05 were considered statistically significant.

## Results

### Genomic characteristics of *L. paracasei* isolated from vaginal flora

**Fig. 1:**
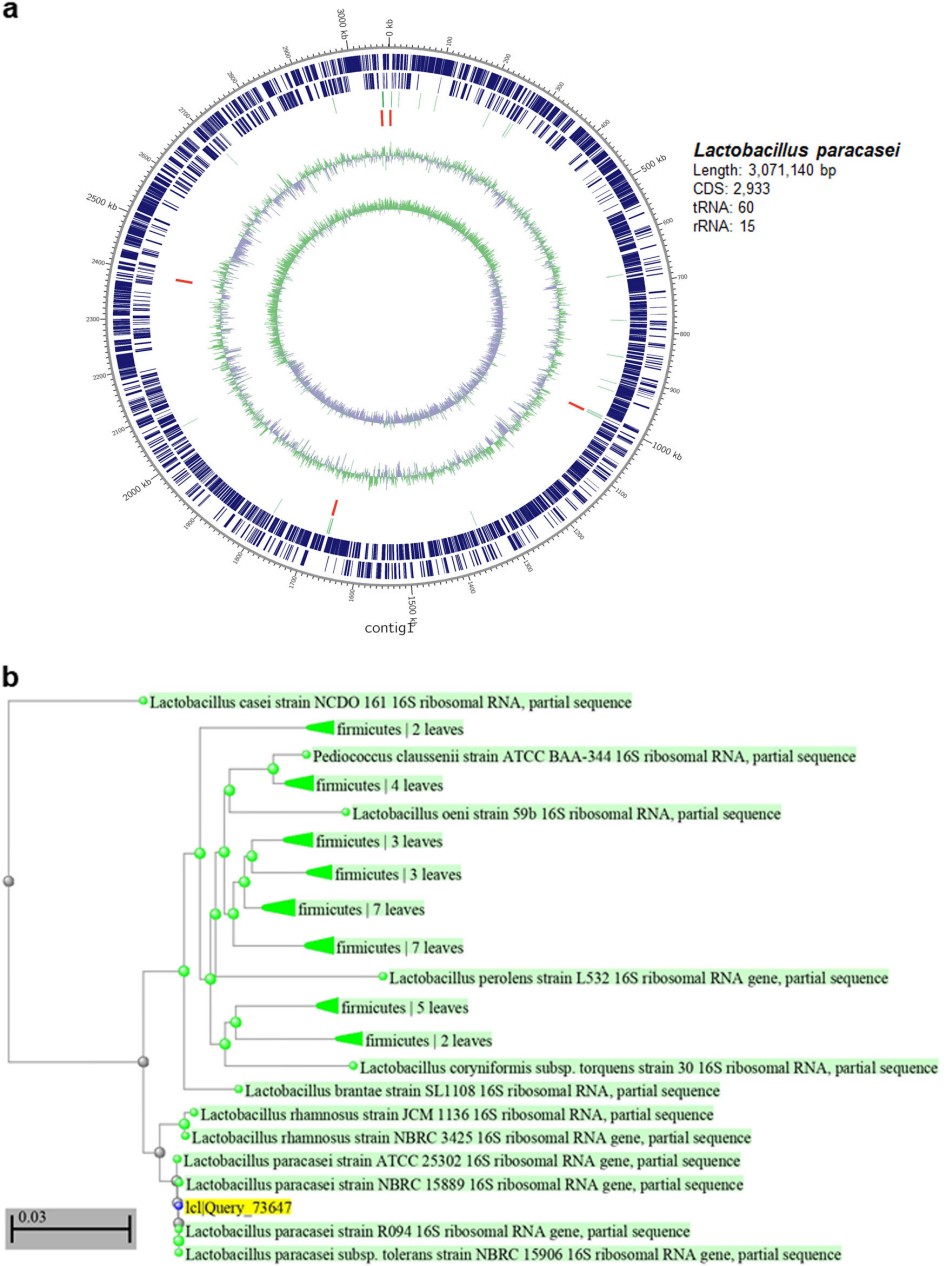
Genomic characteristics of *L. paracasei*. **a** Circular map of *L. paracasei*. The following factors are shown from the outside to the center: CDS (coding sequence) on the forward strand, CDS on the reverse strand, tRNA, rRNA, GC content, and GC skew. **b** Phylogenetic diagram of *L. paracasei*.

Whole-genome sequencing was conducted with the PacBio single-molecule real-time (SMRT) sequencing system to investigate the genome sequence of *L. paracasei* isolated from vaginal flora. De novo assembly utilizing the Hierarchical Genome Assembly Process 3 (HGAP3) software constructed one contig, which included a consensus sequence with higher quality through the self-mapping step. The genome of *L. paracasei* consisted of one circular chromosome (3,071,140 bp), which included 2933 coding sequences, 60 transferRNAs (tRNAs), and 15 rRNAs (Fig. 1a). Phylogenetic analysis based on 16S rRNA revealed that the sequencing pattern of *L. paracasei* used in this study was clustered with other strains within *L. paracasei* (Fig. 1b). Comparisons of the 16S rRNA gene sequence of *L. paracasei* isolated from this study with the corresponding sequence of standard strains from the GenBank database showed that our strain belonged to a subclade of *L. paracasei*. Data from 16S rRNA nucleotide sequence BLAST on NCBI showed that *L. paracasei* used in this study possessed high similarity (99.0–100%) with other strains of *L. paracasei*. Genomic features, BLAST alignment, and comparative results with its close relatives identified the strain used in this study as *L. paracasei* (Supplementary Table 3).

### Isolation and characterization of *L. paracasei*-derived EVs

**Fig. 2:**
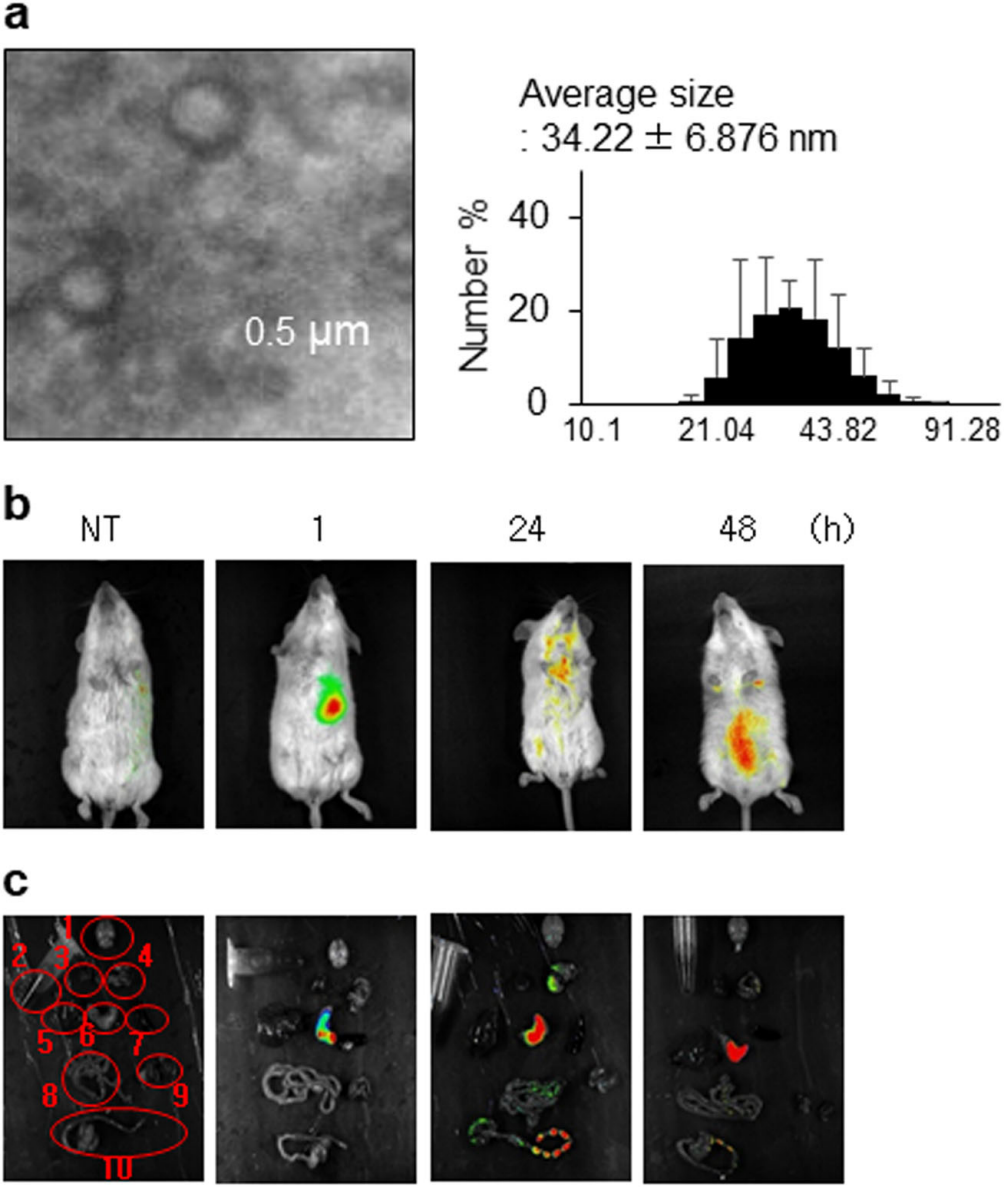
Characterization of *L. paracasei*-derived EV (LpEVs) and targeting of LpEVs to the colon in the mouse model. **a** TEM images (×80,000) of EVs extracted from *L. paracasei*-grown media on day 5. The diameters (nm) of LpEVs measured by nanoparticle tracking analysis (NTA) on day 5 (**b**, **c**) in vivo fluorescence assay. LpEVs were labeled with Cy7-NHS, and 10 µg of Cy7-labeled LpEVs was administered by gavage to the mice. After administration, the fluorescent signal of the whole body or dissected organs was detected depending on the time. 1. brain, 2. blood, 3. heart, 4. lung, 5. liver, 6. stomach, 7. spleen, 8. small intestine, 9. kidney, 10. large intestine.

*L. paracasei*-derived EVs were isolated and purified according to previously described methods^30^. To investigate the characteristics of the LpEVs, EVs purified daily for a week were observed using transmission electron microscopy (TEM) to analyze the morphology of the EVs. TEM images showed the spherical shape of the LpEVs, which consisted of a lipid bilayer. Purified EVs were subjected to dynamic light scattering (DLS) analysis to measure the size distribution of EVs. DLS demonstrated that EV showed a slight variation in diameter ranging from 20 to 100 nm; the average size of EVs was 34.22 ± 6.876 nm (Fig. 2a).

### Effects of LpEVs on LPS-induced inflammatory responses

To evaluate the anti-inflammatory effects of LpEVs, we assessed cell viability and the secretion of tumor necrosis factor-α (TNF-α), a known inflammatory cytokine, in RAW 264.7 murine macrophages treated with EVs derived from *L. paracasei*. No significant differences in cell viability were observed between the negative control and LpEV-treated cells (Supplementary Fig. 1a). Pretreatment with LpEVs inhibited TNF-α secretion in a concentration-dependent manner, while the inhibition of TNF-α secretion was not observed upon pretreatment with *L. paracasei* bacterial pellet (Supplementary Fig. 1b). These results indicate that LpEVs are the most effective in suppressing *E. coli* EV-induced inflammation.

We also evaluated whether LpEVs block NO production in LPS-treated RAW 264.7 cells. LPS induced NO generation at 12 and 24 h, and the levels increased to 83.34% after 12 h of treatment with LPS. As expected, pretreatment of cells with LpEVs reduced LPS-induced NO production (Supplementary Fig. 1c). These data suggest that LpEVs attenuate TNFα secretion accompanying NO production in the inflammatory environment.

### Targeting of LpEVs to the colon in a mouse model

An in vivo imaging study was performed to evaluate whether LpEVs moved to target organs, including the stomach, small intestine, and large intestine, after oral administration. Whole-body imaging showed that LpEVs were present in the stomach areas 1 h after application and diffused in a time-dependent manner (Fig. 2b). In addition, imaging data of dissected organs showed that LpEVs moved from the stomach to the large intestine 24 h after administration and that LpEVs finally disappeared in the large intestine 48 h after application (Fig. 2c).

### LpEVs attenuate LPS-induced inflammation in colon cancer cells

**Fig. 3:**
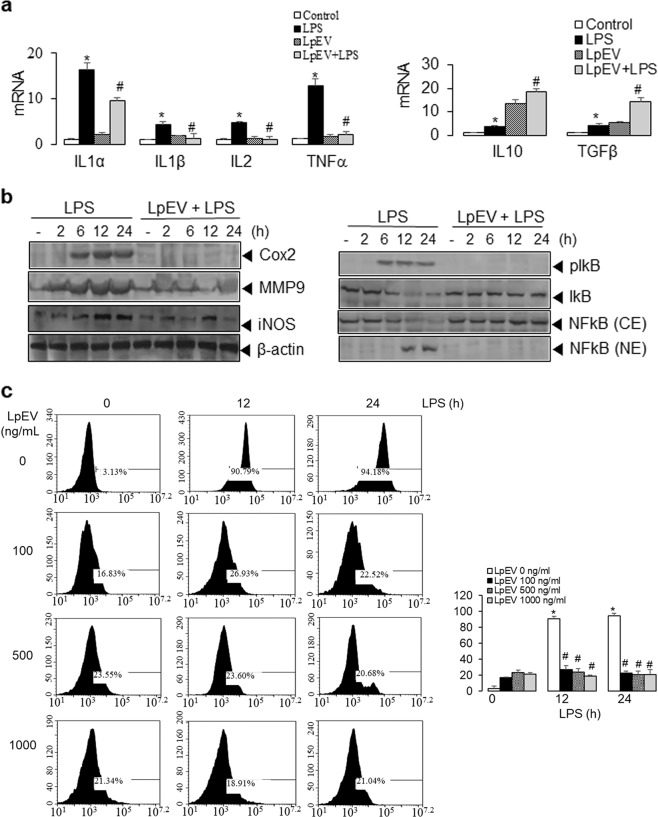
Effects of LpEVs on LPS-induced inflammatory responses in human colorectal cancer cells. **a** HT29 colorectal cancer cells were pretreated with vehicle or 500 ng/mL LpEVs for 12 h and treated with vehicle (control) or 1 mg/mL LPS for 12 h. The mRNA expression of inflammatory cytokines was then analyzed by qRT-PCR, and the percent mRNA expression was plotted as the mean±standard deviation of at least three experiments. **b** HT29 cells were pretreated with vehicle or 500 ng/mL LpEVs for 12 h and then treated with 1 mg/mL LPS for 0, 2, 6, 12, and 24 h. The cells were lysed, and the cell lysates were subjected to SDS-PAGE and western blot analysis using antibodies against COX-2, MMP-9, iNOS, phospho-IκB and IκB, and β-actin. To detect the nuclear translocation of NFκB, cell lysates were fractionated into the nuclear extract (NE) and cytosolic extract (CE) and then analyzed by western blot analysis using an antibody against NFκB p65. **c** The cells were stained with 2,3-diaminonaphthalene for 15 min. NO production was measured by flow cytometry (*N* = 3 for each experimental group). The percentage of NO production was calculated based on naphthalene triazole fluorescence and plotted as the mean±standard deviation of at least three experiments. *P < 0.01 compared with vehicle-treated control cells. ^#^*P* < 0.01 compared with LPS-treated cells without LpEVs.

We examined whether LpEVs inhibit LPS-induced inflammation via in vitro colon cancer cell experiments. Treatment with LPS (1 mg/mL) markedly increased the mRNA levels of the pro-inflammatory cytokines IL-1α, IL-1β, IL-2, and TNFα and slightly increased the mRNA levels of the anti-inflammatory cytokines IL-10 and TGFβ. Pretreatment with LpEVs attenuated the increased expression of pro-inflammatory cytokine mRNAs (IL-1α, IL-1β, IL-2, and TNFα) while enhancing the expression of IL-10 and TGFβ mRNA in HT29 cells treated with LPS (Fig. 3a).

We evaluated whether LpEVs affect LPS-induced inflammation by western blot analysis. LpEVs suppressed the LPS-induced elevated expression of COX-2 and iNOS. In addition, for the NFκB pathway, LPS induced an increase in the phosphorylation of IκB, leading to the degradation and nuclear translocation of NFκB, whereas LpEVs attenuated the LPS-induced activation of these inflammation-associated proteins (the phosphorylation of IκB and the nuclear translocation of NFκB) in HT29 cells (Fig. 3b).

We evaluated whether LpEVs suppress NO production in LPS-treated HT29 cells. LPS induced NO generation at 12 and 24 h, and the levels increased to 90.79% after 12 h of treatment with LPS. As expected, pretreatment of cells with LpEVs reduced LPS-induced NO production (Fig. 3c). These findings show that LpEVs attenuate the expression of inflammatory cytokines and modulators, as well as NO generation, in LPS-treated human colorectal cancer cells.

### LpEVs attenuate the inflammatory response in a DSS-induced colitis mouse model

**Fig. 4:**
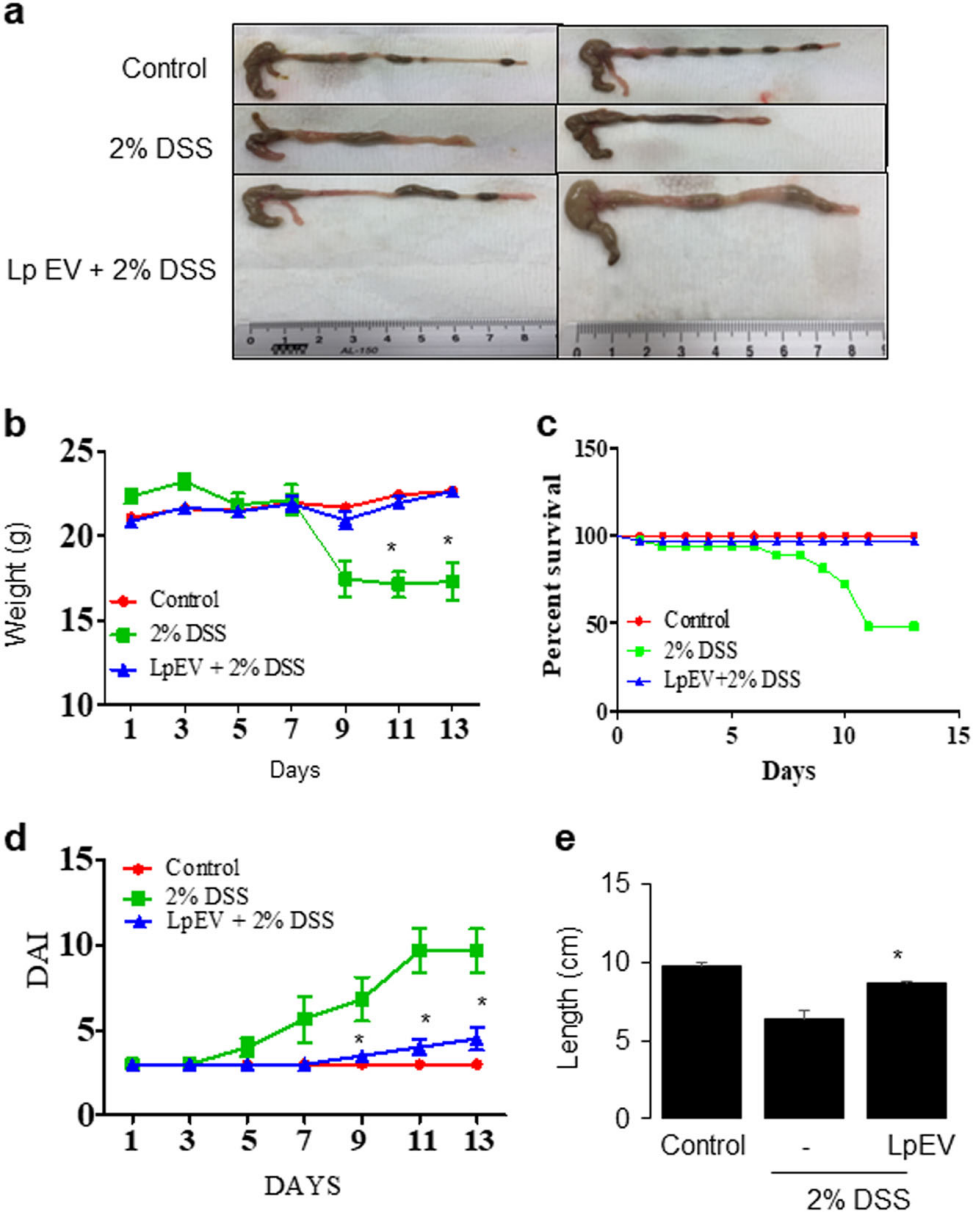
Anti-inflammatory effect of LpEVs in a DSS-induced colitis mouse model. Seven-week-old male C57BL/6 mice were randomly assigned to three groups: the control, 2% DSS-only, and LpEV+2% DSS groups. The mice were orally administered LpEVs (10 mg) in drinking water or 2% DSS for 5 days. **a** Images of colons from each group. **b** Body weight changes in all groups were measured every other day for 2 weeks. **c** Survival ratio. **d** Disease activity index. **e** Colon length. The results are expressed as the mean ± standard deviation of six mice. *P < 0.05 compared with 2% DSS-administered mice without LpEVs.

To examine the in vivo protective effects of LpEVs on acute colitis in mice, 2% DSS was administered to male C57BL/6 mice for 5 days, and then ordinary drinking water was provided for 8 days. The mice were divided into three groups: (1) the control group, which was administered drinking water; (2) the DSS-only group, which was administered 2% DSS in drinking water; and (3) the LpEVs+DSS group, which was administered LpEVs [5 mg/mouse] and 2% DSS in drinking water. To assess the disease activity in each group, body weight loss, survival ratio, colon length at day 13, and DAI were analyzed. The mice in the LpEVs+DSS group showed suppression of body weight loss and mortality increment compared to those of the mice in the DSS group (Fig. 4b, c). Additionally, the LpEVs+DSS group showed a significant decrease in DAI score compared to that of the DSS-only group (Fig. 4d). The mice in the LpEVs+DSS group had longer colon lengths compared to those of the mice in the DSS-only group (Fig. 4a, e). These results suggest that LpEVs significantly attenuate the severity of inflammation in DSS-induced acute colitis mice.

**Fig. 5:**
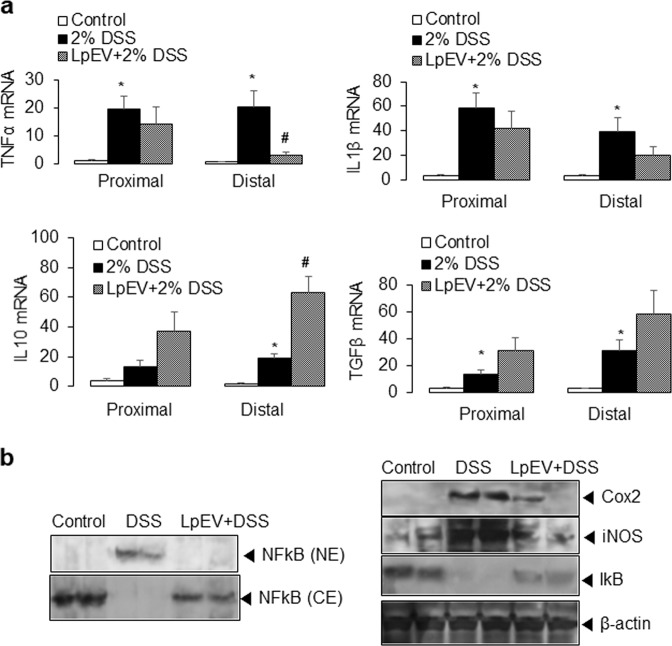
Effect of LpEVs on the expression of inflammatory cytokines and mediators in a DSS-induced colitis mouse model. **a** The mRNA levels of cytokines (TNFα, IL-1β, IL-10, and TGFβ) were analyzed using qRT-PCR. **b** The protein expression of COX-2, iNOS, nuclear NFκB and IκB, and cytosolic NFκB and IκB were analyzed using western blot analysis. *P < 0.05 compared with the drinking water-treated control mice. The results are expressed as the mean±standard deviation of six mice. ^#^*P* < 0.05 compared with the 2% DSS-treated mice without LpEVs.

For cytokine levels, the DSS-only group showed a significant increase in the mRNA levels of the pro-inflammatory cytokines IL-1β and TNFα while showing a slight increase in the mRNA levels of the anti-inflammatory cytokines IL-10 and TGFβ. However, the administration of LpEVs attenuated the increased expression of pro-inflammatory cytokines while further increasing the expression of IL-10 and TGFβ in DSS-induced colitis mice (Fig. 5a). As shown in Fig. 5b, LpEVs attenuated the increased expression of COX-2 and iNOS in colitis mice. In addition, LpEVs reduced the increase in the DSS-induced nuclear translocation of NFκB (Fig. 5b).

### LpEVs activate the unfolded protein response

**Fig. 6:**
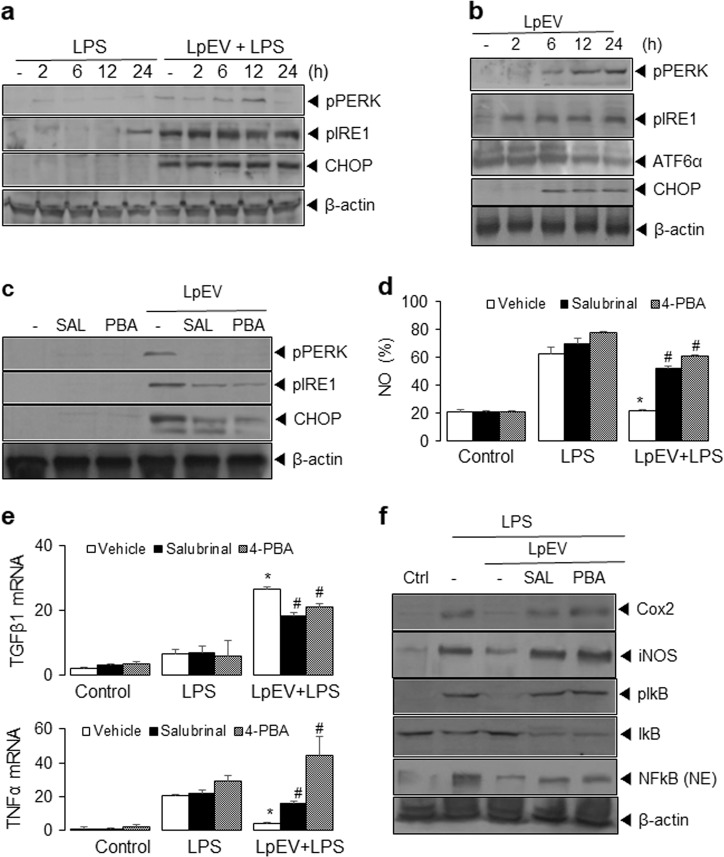
Effect of LpEVs on ER stress-associated proteins in HT29 cells. **a** HT29 cells were pretreated with vehicle or 500 ng/mL LpEVs and then treated with 1 mg/mL LPS for 0, 2, 6, 12, and 24 h. **b** HT29 cells were treated with 500 ng/mL LpEVs for 0, 2, 6, 12, and 24 h. **a**, **b** Cell lysates were resolved by SDS-PAGE and analyzed by western blot analysis with antibodies against CHOP, p-PERK, p-IRE1, ATF6α (p90), and β-actin. **c** HT29 cells were preincubated with 10 µM salubrinal or 5 mM 4-PBA for 1 h and treated with LpEVs for 12 h followed by LPS for 12 h. NO production was determined by a 2,3-diaminonaphthalene assay. **d** HT29 cells were preincubated with 10 µM salubrinal or 5 mM 4-PBA for 1 h, treated with LpEVs for 12 h, and then stimulated with LPS for 12 h. Cell lysates were analyzed by western blot analysis. **e** HT29 cells were preincubated with 10 µM salubrinal or 5 mM 4-PBA for 1 h, treated with LpEVs for 12 h and then stimulated with LPS for 0, 12, and 24 h. The mRNA levels of TNFα and TGFβ were then analyzed by qRT-PCR. **f** HT29 cells were preincubated with 10 µM salubrinal or 5 mM 4-PBA for 1 h, treated with LpEVs for 12 h, and then stimulated with LPS for 0 and 24 h. Cell lysates were analyzed by western blot analysis with antibodies against iNOS, pIκB, IκB, and NFκB. All data were plotted as the mean±standard deviation of at least three experiments. *P < 0.01 compared with LPS-treated cells without LpEVs. ^#^*P* < 0.01 compared with LPS-treated cells with LpEVs.

Because ER stress is thought to participate in LPS-induced inflammation, we investigated whether LpEVs affect the expression of ER stress-associated proteins, such as CHOP, p-PERK, p-IRE1, and cleaved ATF6 utilizing western blot analysis. The results showed that LPS did not alter the unfolded protein response. However, LpEVs significantly induced the expression of CHOP, p-PERK, and p-IRE1 in LPS-treated HT29 cells (Fig. 6a). In addition, LpEVs alone significantly augmented the expression of ER stress-associated proteins (CHOP, p-PERK, p-IRE1, and cleaved ATF6) at 6–24 h (Fig. 6b).

### LpEVs inhibit LPS-induced inflammation via the activation of ER stress

To investigate the relationship between LpEV-induced ER stress and the inhibitory action of LpEVs on the LPS-induced inflammatory response, we used chemical blockers of ER stress: salubrinal, a selective inhibitor of eIF2α dephosphorylation, and 4-PBA, a chemical chaperone. HT29 cells were pretreated with 10 µM salubrinal or 5 mM 4-PBA for 1 h and incubated with LPS for 24 h with or without LpEVs for 12 h. Salubrinal or 4-PBA significantly abrogated the LpEV-induced ER stress response, including CHOP expression, phosphorylation of PERK, and IRE1α in HT29 cells (Fig. 6c). Moreover, salubrinal or 4-PBA markedly reversed the inhibitory effects of LpEVs on the LPS-induced inflammatory response, including NO production (Fig. 6d) and increased TNFα mRNA while decreasing TGFβ mRNA, presumably by inhibiting the expression of ER stress-associated proteins (Fig. 6e). Western blot analysis showed that salubrinal or 4-PBA restored the effects of LPS on the protein expression of COX-2 and iNOS, phosphorylation, the degradation of IκB, and the nuclear translocation of NFκB under inflammation-inhibitory conditions induced by LpEVs (Fig. 6f).

**Fig. 7:**
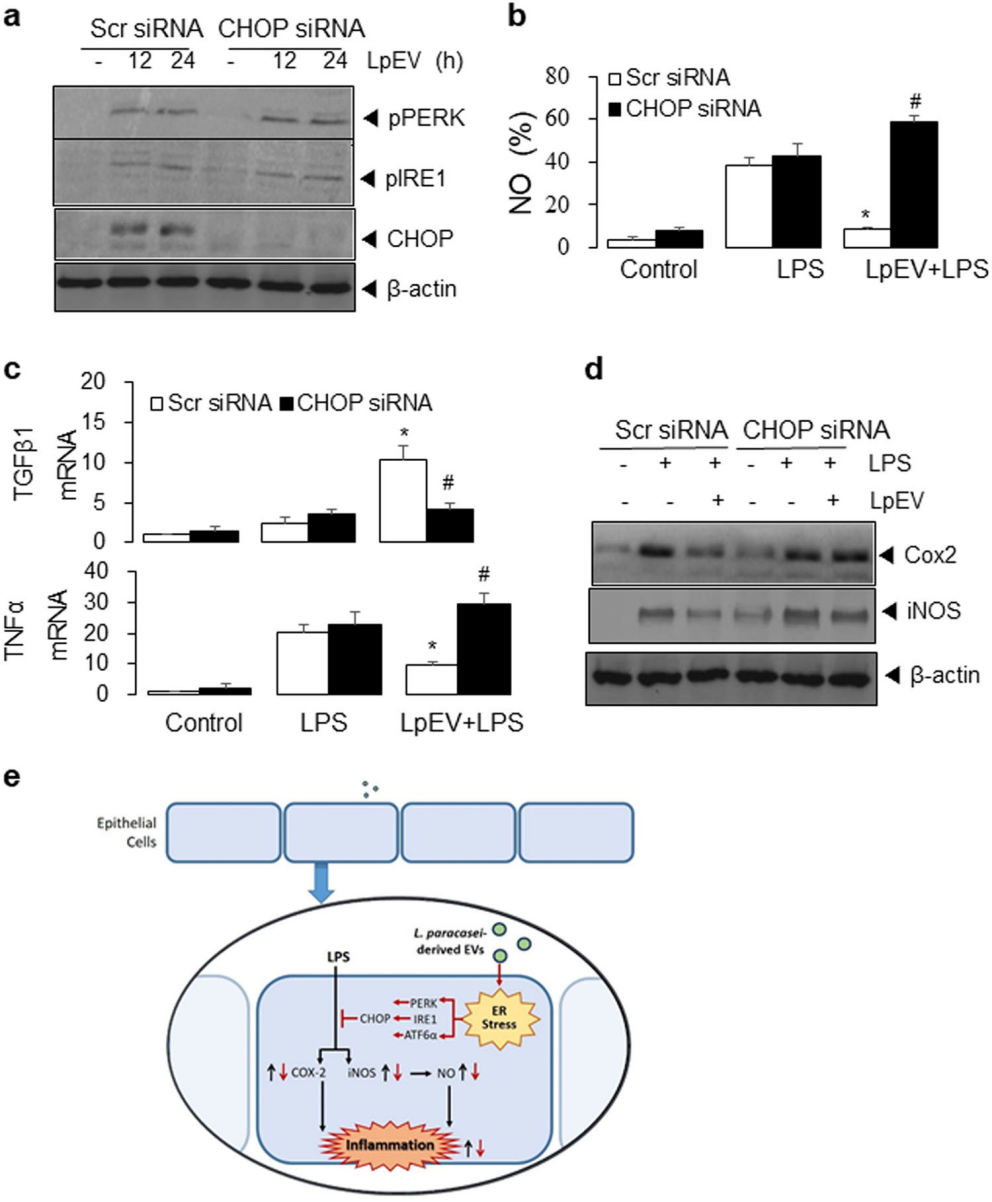
Effect of LpEVs on CHOP expression in HT29 cells. **a** HT29 cells were transfected with scrambled (Scr) control or CHOP siRNA for 24 h and then consecutively treated with LpEVs for 12 h and LPS for 0, 12, and 24 h. NO production was determined by a 2,3-diaminonaphthalene assay. **b** HT29 cells were transfected with scrambled (Scr) control or CHOP siRNA for 24 h and then treated with LpEVs for 0, 12, and 24 h. Cell lysates were resolved by SDS-PAGE and analyzed by western blot analysis. **c**, **d** HT29 cells were transfected with scrambled (Scr) control or CHOP siRNA for 24 h and then consecutively treated with LpEVs for 12 h and LPS for 24 h. **c** The mRNA levels of TNFα and TGFβ were then analyzed by qRT-PCR. **d** Cell lysates were analyzed by western blot analysis with antibodies against COX-2, iNOS, and β-actin. The results shown are representative of those obtained in more than three independent experiments. *P < 0.01 compared with LPS-treated cells without LpEVs. ^#^*P* < 0.01 compared with LPS-treated cells with LpEVs. **e** The proposed model of LpEV-induced ER stress against LPS-induced inflammation in HT29 human colorectal cells. LPS induces COX-2, iNOS, and NFκB activation, resulting in NO production and inflammatory cytokine activation. LpEVs increase the ER stress response and the activation of PERK, IRE1, and ATF6α, leading to CHOP expression, which attenuates LPS-induced inflammatory responses.

To confirm the role of ER stress and CHOP expression in the inhibitory effects of LpEVs on the inflammatory response induced by LPS, we suppressed CHOP expression by CHOP siRNA transfection for 24 h and examined the effect of LpEVs on LPS-induced inflammatory responses at 24 h. The knockdown of CHOP did not significantly reduce the expression of unfolded response proteins (the phosphorylation of PERK and IRE1α) except CHOP in cells treated with LpEVs (Fig. 7a). However, CHOP knockdown significantly reversed the inhibitory effects of LpEVs on the LPS-induced inflammatory response, including NO production (Fig. 7b) and increased TNFα mRNA, while decreasing TGFβ mRNA (Fig. 7c). Western blot analysis showed that CHOP siRNA transfection abrogated the inhibitory effects of LpEVs on the LPS-induced protein expression of COX-2 and iNOS (Fig. 7d). These results suggest that LpEV-induced CHOP expression might be responsible for the inhibition of LPS-induced inflammation. Collectively, these findings suggest that LpEVs attenuate LPS-induced intestinal inflammation via activating ER stress (Fig. 7e).

## Discussion

In this study, we demonstrated that LpEVs have an anti-inflammatory effect by regulating the expression of cytokines and inflammatory mediators as well as NO generation. Consistent with the in vitro results, LpEVs showed protective properties in a DSS-colitis mouse model. Furthermore, ER stress is one of the major mechanisms of action of LpEV-mediated inflammatory control under LPS treatment in HT29 human colorectal cancer cells.

Previously, it was demonstrated that *Lactobacillus* spp. exert a suppressive effect on various inflammatory disorders^31–34^. *L. rhamnosus* (4B15) and *L. gasseri* (4M13) have been reported to have antioxidative activity, inhibit α-glucosidase activity, reduce cholesterol, and suppress NO production. In addition, two strains significantly block the release of inflammatory cytokines, including TNFα, IL-6, IL-1β, and IL-10, in LPS-treated RAW 264.7 cells^35^. *L. acidophilus* blocks the colitis-associated immune response of the IL-23/Th17 axis by downregulating the activity of IL-17, TNFα, IL-23, TGFβ1, and STAT3^36^. *L. paracasei* has been shown to induce anti-inflammatory responses in IBD, including chemically and pathogenically induced colitis models^37–39^. Moreover, *L. paracasei* has improved anticancer effects on colon tumorigenesis^40,41^. Furthermore, it was previously shown that *L. plantarum*-derived EVs suppress inflammatory responses in *S. aureus*-induced atopic dermatitis mice by blocking the secretion of IL-6 and IL-4^18^. In addition, EVs isolated from mouse serum and fed *L. plantarum* and *L. rhamnosus* inhibit the production of TNFα and IL-6 in LPS-treated RAW 264.7 mouse macrophages^42^.

Collectively, these findings support the anti-inflammatory capability of various *Lactobacillus* spp. as well as their secreted EVs. Interestingly, the particular *Lactobacillus* spp. used in this study, *L. paracasei*, was isolated from the vagina rather than the gut microbiota. At birth, gestational flora influence the immune development of the early gut flora, and the microbiota initially populated primarily with *Lactobacillus*-dominant flora is obtained at birth through the vaginal canal^4,43^. Therefore, great interest lies in the probiotic capabilities of Lactobacilli from healthy vaginal flora not only for the treatment of vaginal dysbiosis but also for the treatment of gastrointestinal illness. This study provides evidence of the efficacy of EVs derived from beneficial vaginal bacterial species in the treatment of gastrointestinal inflammatory conditions, revealing not only the probiotic anti-inflammatory capabilities of *L. paracasei* but also the potential relationship between EVs secreted from the vaginal microbiota and gastrointestinal health. Future studies should be conducted to further elucidate the relationship between EVs secreted from the vaginal flora and the immune response in gastrointestinal diseases such as IBD.

At the molecular level, ER stress response modification was found to be the primary mechanism by which LpEVs were able to induce an anti-inflammatory response. Nitric oxide synthase (NOS) is primarily responsible for NO production in mammals, and inducible nitric oxide synthase (iNOS, NOS2) is activated by inflammatory cytokines, endotoxins, and a hypoxic environment^44^. COX-2 is an enzyme that catalyzes prostaglandin production from arachidonic acid and functions in inflammation. Bacterial endotoxins, LPS, cytokines, growth factors, and hormones stimulate COX-2 activation, resulting in the inflammatory response^45^. COX-2 regulates iNOS expression and vice versa^46,47^. NFκB is known as the primary regulator of iNOS and COX-2 (refs. ^48,49^) and is considered to be the critical transcription factor in inflammatory responses induced by multiple cytokines and pathogens^50^. In this study, we showed that while LPS increased the expression of COX-2 and iNOS and the nuclear translocation of NFκB, the administration of LpEVs was able to reduce the associated LPS-induced inflammatory responses in HT29 cells.

NO plays a crucial role in the signal transduction pathway involved in cell proliferation, survival, and cell death in almost all types of cells^51,52^. Additionally, NO is a well-known critical factor involved in inflammatory responses in many types of cells, including macrophages^53,54^. The abnormal generation of NO induces an inflammatory response that is potentially toxic to adjacent cells and host tissues. Of all the inflammatory mediators, NO is considered to be the most active mediator in colorectal cancer development and mainly associated with the severity of IBD^55,56^. In the present study, we found that LPS-induced NO generation in HT29 cells and LpEVs reduced NO levels that were elevated in LPS-treated cells. These findings suggest that LpEVs inhibit LPS-induced NO production by suppressing iNOS activation.

Furthermore, LpEVs induced the activation of ER stress-associated proteins, stimulating the phosphorylation of PERK and IRE1, ATF6 cleavage, and CHOP expression. The suppression of ER stress by the chemical inhibitors salubrinal and 4-PBA and siRNA targeting CHOP enhanced the LPS-induced expression of COX-2 and iNOS, the transcriptional activation of inflammatory cytokines, and NO production. These data suggest that ER stress might be involved in the inhibitory effects of LpEVs on LPS-induced inflammatory responses in human colorectal cancer cells.

In conclusion, the present study found that LpEVs induce ER stress, which contributes to the suppressive effects on LPS-mediated intestinal inflammation via COX-2, iNOS, and NFκB. We also confirmed the anti-inflammatory effect of LpEVs on acute colitis using a mouse model. For the first time, we found that ER stress is a major mechanism involved in the anti-inflammatory effects of LpEVs against LPS- and DSS-mediated inflammation. Further studies are required to determine the anti-inflammatory effects and mechanisms of action of LpEVs in vitro and in vivo and the potential of LpEVs as novel anti-inflammatory agents for IBD.

## Supplementary information

Supplementary information
