# Supplementary material for: Lactobacillus paracasei-derived extracellular vesicles attenuate the intestinal inflammatory response by augmenting the endoplasmic reticulum stress pathway
Source: Exp Mol Med. 2020 Mar 2;52(3):423–37. doi: 10.1038/s12276-019-0359-3 (PMC7156483; doi:10.1038/s12276-019-0359-3)

Supplementary Table 1. Mouse primers of cytokines

	Forward primer	Reverse primer
IL-1β	GAGCCCATCCTCTGTGACTC	TCCATTGAGGTGGAGAGCTT
IL-10	CAGCCGGGAAGACAATAACT	TCATTTCCGATAAGGCTTGG
TGFβ	CCTGCAAGACCATCGACATG	TGTTGTACAAAGCGAGCACC
TNFα	ACGGCATGGATCTCAAAGAC	AGATAGCAAATCGGCTGACG
β-actin	CATCCGTAAAGACCTCTATGCCAAC	ATGGAGCCACCGATCCACA

Supplementary Table 2. Human primers of cytokines

	Forward primer	Reverse primer
IL-1α	TCAAGGAGAGCATGGTGGTA	GTGCTGACCTAGGCTTGATG
IL-1β	AGCTCGCCAGTGAAATGATG	CGGAGATTCGTAGCTGGATG
IL-2	AACCTCTGGAGGAAGTGCTA	AATGGTTGCTGTCTCATCAGC
IL-10	CCTGCCTAACATGCTTCGAG	GTCTTGGTTCTCAGCTTGGG
TGFβ	GACTCGCCAGAGTGGTTATC	GGTAGTGAACCCGTTGATGT
TNFα	ATCATCTTCTCGAACCCCGA	GTTATCTCTCAGCTCCACGC
β-actin	TGACGGGGTCACCCACACTG	AAGCTGTAGCCGCGCTCGGT

Supplementary Table 3. Alignment results of *L. paracasei*

Subject acc.	% identity	Alignment length	Mismatches	Q. start	Q. end	S. start	S. end	E value	Bit score
Lactobacillus paracasei strain R094 16S ribosomal RNA gene, partial sequence	100<br>99.934<br>99.869<br>99.869<br>100<br>98.98	1522<br>1522<br>1522<br>1522<br>1326<br>196	0<br>1<br>2<br>2<br>0<br>2	999528<br>2389192<br>1668500<br>3051581<br>1<br>3070945	1001049<br>2390713<br>1670021<br>3053102<br>1326<br>3071140	1522<br>1<br>1522<br>1<br>197<br>1	1<br>1522<br>1<br>1522<br>1522<br>196	0<br>0<br>0<br>0<br>0<br>1.00E-92	2811<br>2806<br>2800<br>2800<br>2449<br>351
Lactobacillus paracasei strain NBRC 15889 16S ribosomal RNA gene, partial sequence	99.933<br>99.866<br>99.799<br>99.799<br>99.923<br>98.98	1495<br>1495<br>1495<br>1495<br>1299<br>196	1<br>2<br>3<br>3<br>1<br>2	999555<br>2389192<br>1668527<br>3051581<br>1<br>3070945	1001049<br>2390686<br>1670021<br>3053075<br>1299<br>3071140	1495<br>1<br>1495<br>1<br>197<br>1	1<br>1495<br>1<br>1495<br>1495<br>196	0<br>0<br>0<br>0<br>0<br>1.00E-92	2758<br>2752<br>2747<br>2747<br>2396<br>351
Lactobacillus paracasei subsp. tolerans strain NBRC 15906 16S ribosomal RNA gene, partial sequence	99.866<br>99.799<br>99.733<br>99.733<br>99.846<br>98.974	1496<br>1496<br>1496<br>1496<br>1301<br>195	0<br>1<br>2<br>2<br>0<br>2	999555<br>2389193<br>1668527<br>3051582<br>1<br>3070946	1001048<br>2390686<br>1670020<br>3053075<br>1299<br>3071140	1497<br>2<br>1497<br>2<br>197<br>2	2<br>1497<br>2<br>1497<br>1497<br>196	0<br>0<br>0<br>0<br>0<br>3.60E-92	2750<br>2745<br>2739<br>2739<br>2390<br>350

Lactobacillus paracasei strain ATCC 25302 16S ribosomal RNA gene, partial sequence	99.792<br>99.723<br>99.653<br>99.653<br>99.921<br>97.714	1442<br>1442<br>1442<br>1442<br>1267<br>175	1<br>2<br>3<br>3<br>1<br>2	999587<br>2389214<br>1668559<br>3051603<br>1<br>3070967	1001027<br>2390654<br>1669999<br>3053043<br>1267<br>3071140	1441<br>1<br>1441<br>1<br>175<br>1	1<br>1441<br>1<br>1441<br>1441<br>174	0<br>0<br>0<br>0<br>0<br>3.67E-77	2645<br>2639<br>2634<br>2634<br>2335<br>300

Supplementary Fig. 1. Effects of LpEVs on *E.coli* EV-induced inflammatory responses in murine macrophages

(a) Cytotoxicity of RAW 264.7 cells (b) TNFα secretion (c) NO production (N=3 for each experimental group). $^{*}P<0.01$ compared with vehicle-treated control cells. $^{\#}P<0.01$ compared with LPS-treated cells without LpEVs.

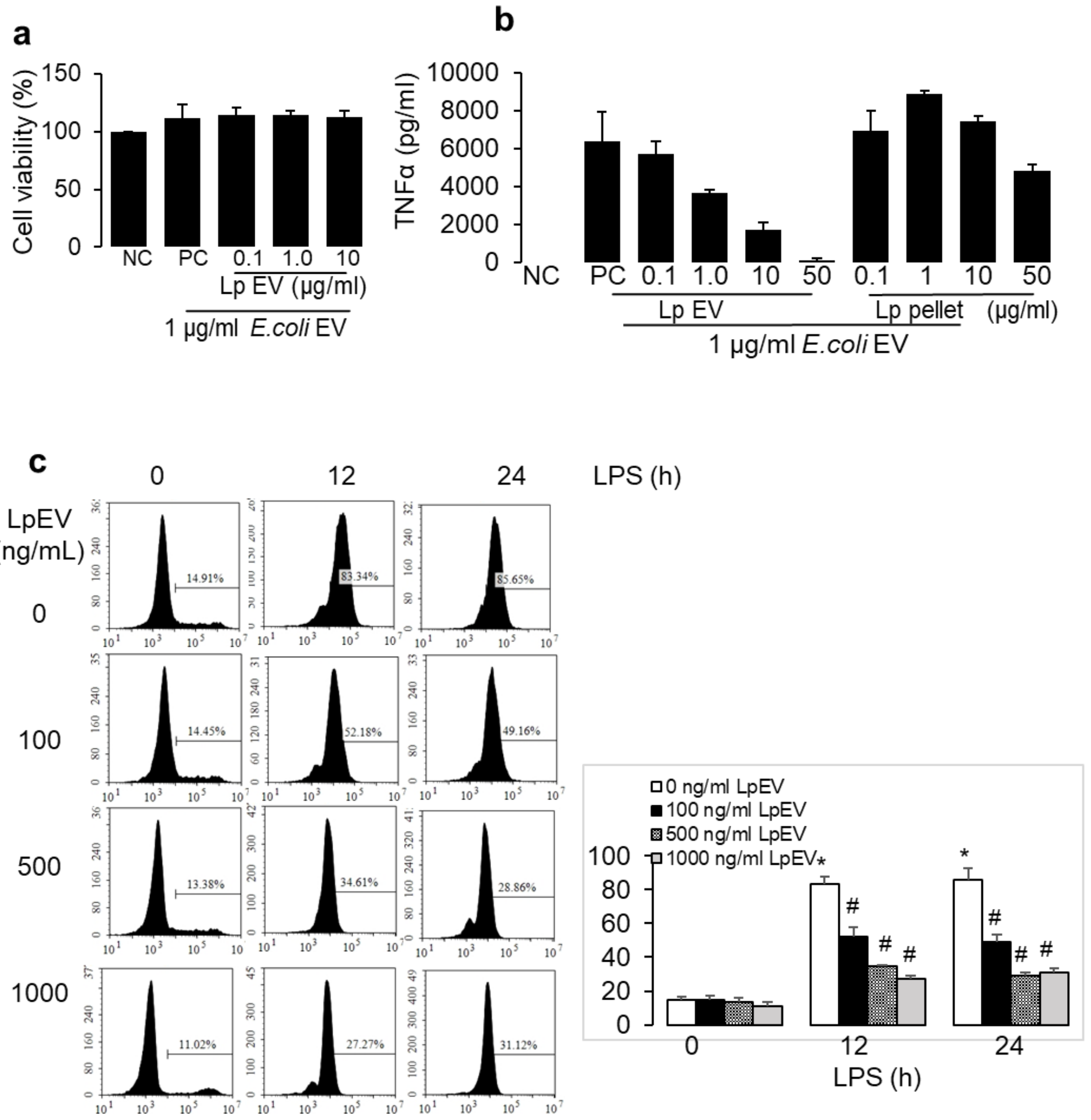

Supplement: Supplementary file 1 — Supplementary information [file 12276_2019_359_MOESM1_ESM.pdf]
